# What Is Addiction?

**Published:** 2008

**Authors:** Henry R. Kranzler, Ting-Kai Li

**Keywords:** Addiction, alcohol and other drug (AOD) use, abuse and dependence, AOD use disorders (AODUDs), substance abuse, substance dependence, *Diagnostic and Statistical Manual of Mental Disorders (DSM)*

## Abstract

This issue of *Alcohol Research & Health* examines addiction to multiple substances—that is, combined dependence on alcohol and other drugs (AODs), including marijuana, cocaine, and opioids. It seems fitting, then, to begin the issue with a look at what constitutes “addiction.” The *Oxford English Dictionary* (pp. 24–25) traces the term addiction to Roman law, under which addiction was a “formal giving over by sentence of court; hence, a dedication of person to a master.” This notion of relinquishment of control by the addicted person is the central feature of many lay and professional definitions of the term. The study of addictive behavior crosses several disciplines, including, among others, behavioral neuroscience, epidemiology, genetics, molecular biology, pharmacology, psychology, psychiatry, and sociology. Articles in this issue examine aspects of AOD use disorders from the perspective of some of these varied disciplines.

The term “substance dependence” has gained great currency because of its use in the *Diagnostic and Statistical Manual of Mental Disorders* (DSM). The DSM, both in its revision of the third edition (DSM–III–R; American Psychiatric Association [APA] 1987) and in its most recent edition (DSM– IV; APA 1994), avoids the term addiction, preferring instead to use the diagnoses of substance abuse and dependence, collectively referred to as substance use disorders. Beginning with DSM–III–R, the criteria used to diagnose substance use disorders were applied more or less equally to all of the substances that are commonly misused by individuals. In the DSM, therefore, individuals are differentiated into three mutually exclusive categories: no substance use disorder, abuse only, or dependence. With this approach, abuse is diagnosed only if the individual does not meet the criteria for dependence. Accordingly, an individual meeting the criteria for both abuse and dependence is diagnosed only with dependence. The most recent text revision of the DSM (DSM– IV–TR; APA 2000, p. 192) identifies impaired control over substance use as the essential feature of dependence, which is “a cluster of cognitive, behavioral, and physiological symptoms indicating that the individual continues use of the substance despite significant substance-related problems.”

The dependence syndrome, which forms the basis for the diagnostic approach used in DSM–III–R, was first described for alcohol by Edwards and Gross (1976); it was later broadened to include other drugs (Edwards et al. 1981). However, as was true for DSM–III–R (APA 1987), the inclusion of abuse as a distinct category in DSM–IV deviated from the purely dimensional approach (in which all dependence occurs on a continuum, varying from no dependence symptoms to severe dependence) taken by Edwards and colleagues. This dimensional approach recently has been supported by findings from a large, nationally representative sample of more than 43,000 people. Saha and colleagues (2006) found that, except for alcohol-related legal problems, all DSM–IV criteria for alcohol abuse and dependence formed a continuum of alcohol use disorder severity. Moreover, only one of four diagnostic criteria for alcohol abuse (i.e., hazardous use) fell among other criteria associated with mild dependence, whereas the other three abuse criteria clustered with the most severe symptoms of dependence. These findings call into question the distinction between abuse and dependence and the identification of abuse as being milder than dependence.

O’Brien and colleagues (2006) have argued against the use of the term substance dependence, calling for a renewed emphasis on addiction. Dependence, they pointed out, is often confused with physical dependence (i.e., the adaptations that result in withdrawal symptoms when substance use is discontinued), which can occur with therapeutic applications of a variety of medications. This terminological confusion may make clinicians reluctant to prescribe pain medications, for example, for fear of causing addiction. By emphasizing the behavioral aspects of compulsive substance use, addiction captures the chronic, relapsing, and compulsive nature of substance use that occurs despite the associated negative consequences. On that basis, these authors urged the APA to restore the use of the term addiction in the DSM–V, which currently is in development. A disadvantage of the term addiction, however, is that it often is used pejoratively and can lead practitioners to avoid its use for fear of stigmatizing their patients and damaging their relationship with them. Further, the term addiction has been used so widely and variably that, like “alcoholism,” its meaning has been diluted, substantially limiting its value.

The terminology used to describe alcohol and other drug (AOD) use disorders (AODUDs) is of key importance to both the study and the clinical care of people suffering from these conditions. AODUDs result from a combination of genetic, environmental, social, and psychological factors. The heterogeneity of addictive disorders is well recognized, with a key dimension of subgroups being the pattern of dependence on multiple substances (Babor et al. 1992; Kranzler et al. 2008). The study of addictive behavior therefore crosses several disciplines, including behavioral neuroscience, epidemiology, genetics, molecular biology, pharmacology, psychology, psychiatry, and sociology. Terminological distinctions have become all the more important with the growing emphasis on multidisciplinary research and treatment approaches, as researchers and clinicians must be able to communicate effectively across disciplines. Articles in this issue of *Alcohol Research & Health* examine aspects of AODUDs from the perspective of some of these varied disciplines. However, given the pros and cons of the use of the terms dependence and addiction, as well as the absence of a clear consensus on which is preferred, in this volume the terms are used more or less interchangeably.

Four articles in this volume focus on the etiology and development of AODUDs. The article by Drs. Dick and Agrawal discusses both genetic and environmental risk factors for AOD use and for co-occurring dependence on AODs. In a related article, Dr. Wand describes how stress and the genetics of the stress response influence risk for dependence. Dr. Cruz and colleagues’ article reviews the neurobiological mechanisms of addiction and how they may influence co-morbid AOD use. Drs. Thatcher and Clark review psychological risk factors for adolescent AOD use, including parental substance use disorders and psychological dysregulation; child deficits in cognitive, behavioral, and emotional regulation that lead to experimentation with AODs; and the development of substance-related problems.

Other articles in this issue focus on the epidemiology and timing of AOD use. Dr. Falk and colleagues report on the co-morbidity of AOD use and disorders in the general population. Dr. Martin distinguishes between the use of alcohol at the same time as other drugs and alcohol consumption that occurs separately from other drug use.

The diagnosis and treatment of AODUDs are covered separately in this issue. In the first of two articles, Drs. Arnaout and Petrakis describe challenges to the diagnosis of AODUDs and factors—such as the patient’s gender, family history, and course of illness over time—that influence the accuracy of diagnosis. The second article, by Drs. Arias and Kranzler, examines the literature on the treatment of patients with co-morbid AOD dependence, including concurrent and sequential treatment approaches, psychotherapy and pharmacotherapy, and treatment matching.

Clearly, AOD use, abuse, and dependence are far-reaching phenomena. Similar to the concept of craving, which seeks to provide an experiential basis to explain compulsive drug use but which has limited precision, the meaning of the terms substance abuse, dependence, and addiction will continue to evolve and must be further validated empirically. Refining these terms is central to helping researchers and clinicians understand more fully the phenomena to which they refer and to develop methods to diagnose and treat co-morbid AODUDs effectively. Despite the lack of consensus on terminology, we hope that this issue of *Alcohol Research & Health* adequately presents the literature on the prevalence, complex mechanisms, adverse consequences, and challenges to the evaluation and treatment of co-occurring use, abuse, and dependence on AODs.
